# Establishing a reliable framework for harnessing the creative power of the scientific crowd

**DOI:** 10.1371/journal.pbio.2001387

**Published:** 2017-02-15

**Authors:** Adrian J. Carter, Amy Donner, Wen Hwa Lee, Chas Bountra

**Affiliations:** 1 Department of Discovery Research Coordination, Boehringer Ingelheim, Ingelheim, Germany; 2 The Chemical Probes Portal, Genetics Medicine Research Building, Chapel Hill, North Carolina, United States of America; 3 Structural Genomics Consortium, Nuffield Department of Clinical Medicine, University of Oxford, Oxford, United Kingdom

## Abstract

Discovering new medicines is difficult and increasingly expensive. The pharmaceutical industry has responded to this challenge by embracing open innovation to access external ideas. Historically, partnerships were usually bilateral, and the drug discovery process was shrouded in secrecy. This model is rapidly changing. With the advent of the Internet, drug discovery has become more decentralised, bottom-up, and scalable than ever before. The term open innovation is now accepted as just one of many terms that capture different but overlapping levels of openness in the drug discovery process. Many pharmaceutical companies recognise the advantages of revealing some proprietary information in the form of results, chemical tools, or unsolved problems in return for valuable insights and ideas. For example, such selective revealing can take the form of openly shared chemical tools to explore new biological mechanisms or by publicly admitting what is not known in the form of an open call. The essential ingredient for addressing these problems is access to the wider scientific crowd. The business of crowdsourcing, a form of outsourcing in which individuals or organisations solicit contributions from Internet users to obtain ideas or desired services, has grown significantly to fill this need and takes many forms today. Here, we posit that open-innovation approaches are more successful when they establish a reliable framework for converting creative ideas of the scientific crowd into practice with actionable plans.

## Cooperation and competition

Discovering a new medicine is difficult, yet the future of the pharmaceutical industry depends on its ability to create medicines that provide benefit to patients. Although the United States Food and Drug Administration approved a record number (45) of new drugs in 2015 [1], overall, clinical success rates remain low, with roughly only one in ten (10.4%) clinical drug candidates reaching the market. Furthermore, half of the 2015 drugs were approved via the orphan drug process, thereby suggesting that the industry is trending towards specialty indications [2]. Even more worrying is that overall phase III success rates are only 50% [3]. Indeed, this last-named study noted that half of the suspensions of phase III trials were attributable to lack of efficacy, which indicates that the processes companies use to discover and select clinical targets are functioning poorly. Discovering new medicines is also becoming more expensive. A study of the research and development (R&D) costs of 106 randomly selected new drugs from ten pharmaceutical firms demonstrated that the estimated average out-of-pocket, capitalised expenses per approved new compound amount to $2,558 million, measured in 2013 US dollars [4]. This equates to an annual rate of increase of 8.5% over the rate of inflation compared with previous studies from earlier time periods and, according to the authors, may still be an underestimate. These studies clearly indicate how expensive and difficult it is to develop a new medicine today.

At the same time, public funding bodies are tightening their belts and setting new priorities such that academic institutions across the world are facing a decrease or redistribution of their research funding [5]. Consequently, the governing councils of academic institutions and hospitals are turning to the private sector for more funding. The private sector is also relying more on the public sector to derisk new drug discovery approaches. The challenge for all those involved (government, charitable, philanthropic, and private funders, biotech industry, pharmaceutical industry, academia, and patient groups) is to identify those opportunities with the greatest potential to provide patient and societal benefit and align their resources to achieve maximal and rapid impact. To meet this challenge, we must explore the best ways to optimise the processes by which academia and industry work together to solve such fundamental scientific and medical problems in the process of making new medicines. The best processes likely involve a combination of cooperation and competition. Pharmaceutical rivals are now cooperating in the early stages of discovery research in precompetitive public–private partnerships (PPPs) to access the expertise of the global biomedical community [6]. Many of these companies are also opening competitions to bring new minds and skillsets to bear on problems in biomedical research via crowdsourcing initiatives [7]. We believe that collaborative PPPs and competitive crowdsourcing can improve the process of target discovery and selection and that improvements in this step will accelerate the creation of new medicines for patients.

## Opening the innovation process

**Fig 1:**
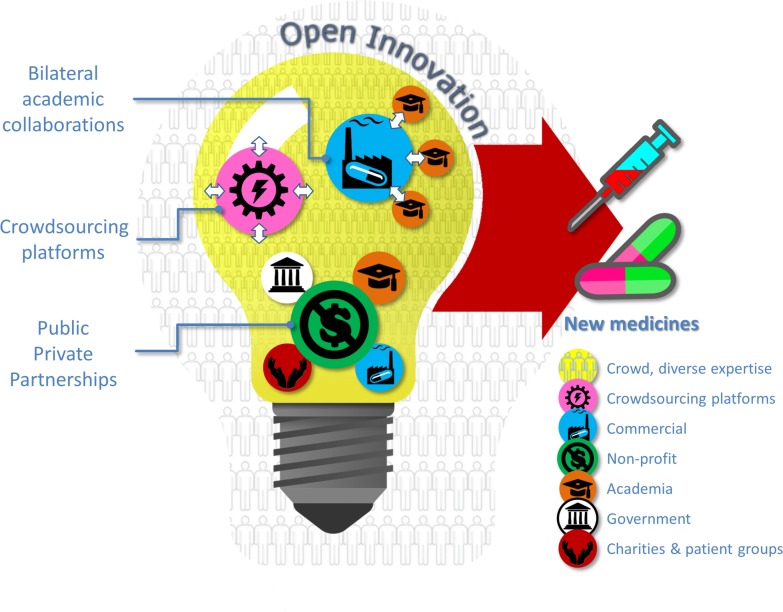
Lighting up the discovery of new medicines with the power of the crowd. Bilateral academic collaborations, crowdsourcing platforms, and PPPs are different approaches for bringing together industry, non-profit organisations, academic scientists, government bodies, charities, and patient groups in different combinations in order to identify bigger and brighter ideas for new medicines based on the power of the scientific crowd.

There are several ways to open up innovation (Fig 1). Historically, collaborations in the health care business between the public and private sectors have been bilateral agreements between one pharmaceutical company and one principal investigator and/or scientific institution. The scientific reputation of the collaborators, their background patent rights, or a recent scientific discovery typically drove the formation of such collaborations. Indeed, such one-to-one partnership models formed the basis for the definition of “open innovation” provided by Henry Chesbrough [8] when he said that companies should use external as well as internal ideas to advance their technology. The term open innovation is now accepted as just one of many terms that capture different but overlapping types of openness during the innovation process [9]. Open initiatives have all been revolutionised by the Internet and the World Wide Web. In the nonnetworked world, before the Internet, innovation was a centralised, top-down, and difficult-to-scale-up process; in contrast, open innovation in the networked world is increasingly decentralised, bottom-up, and scalable [10]. The best example of this evolution is represented by the story of the GNU Project and Linux. GNU is a recursive acronym for "GNU's Not Unix”, and the GNU Project was a mass collaboration software project announced in 1983 by Richard Stallman [11]. His aim was to give computer users freedom and control in their use of their computers and computing devices by collaboratively developing and providing software. Stallman wrote the accompanying GNU general purpose license, which guarantees end users the freedom to run, study, share (copy), and modify the software. The original Linux kernel, or core of the system that manages the central processing unit, memory, and peripheral devices, was conceived and created in 1991 by Linus Torvalds as an operating system (OS) for his personal computer. The combination of GNU software and the Linux kernel, commonly known as GNU/Linux, is now used as the basis for various embedded devices such as routers, wireless access points, set-top boxes, smart televisions, and, perhaps most famously, as the basis for the Android OS for tablet computers and smartphones [11]. Software companies have recognised that by revealing some of their internally developed code within embedded Linux, they have been able to benefit from community-based development support: smaller companies appear to disclose more software code than larger ones, and yet, on average, companies reveal about half of the code they have developed for embedded Linux, i.e., they reveal selectively [12].

In order to access the wider scientific community, the pharmaceutical industry is coming to appreciate the advantages of selective revealing in the same way that the software industry does [13]. Selective revealing refers to a situation in which companies consciously decide to disclose their proprietary information in the expectation that they will receive valuable information in the future. The process comes in two major forms: solutions in the form of tools or problems revealed as questions. In a precompetitive PPP, private and public funders agree to define areas that would benefit from a joint research programme and share ideas, protocols, and tools; crowdsourcing, on the other hand, calls for companies to admit to the wider scientific community what they do not know in order to solicit possible solutions. The number and scope of precompetitive PPPs have steadily been increasing because pharmaceutical rivals increasingly want to cooperate in the early stages of discovery research to accelerate and improve the efficiency of the process [6]. In PPPs, several companies work with one or more public institutions in defined areas whilst agreeing to share all results with a wider audience without restriction [14]. Although management of such entities is challenging [15], there have been several examples of successful consortia: the Single Nucleotide Polymorphisms Consortium [16], the International HapMap Project [17], and the Structural Genomics Consortium (SGC) [18]. Indeed, the SGC’s epigenetics programme (see below) provides a prime example of what can be achieved by such an open consortium in drug discovery, thereby demonstrating that open innovation can be integrated into a traditional pharmaceutical R&D model and that it should not be regarded as a substitute but as a complement.

## High-quality chemical tools help

Targeting epigenetic proteins is a rapidly growing area for medicinal chemistry and drug discovery [19]. In 2010, the SGC reported a cell-permeable, high-quality chemical probe for the bromodomain and extra-terminal (BET) family member BRD4, JQ1, which has expanded our understanding of the role of this epigenetic reader protein in certain forms of human squamous carcinoma [20]. With the help of JQ1 and an RNA interference screen, scientists have subsequently shown that BRD4 is also a potential therapeutic target for the treatment of acute myeloid leukaemia [21]. Importantly, JQ1 was made freely available to the broader scientific community. Because of this availability, the academic crowd facilitated the discovery and dissemination of biological knowledge on BRD4, which was a novel target when JQ1 was discovered. The scientific crowd has now demonstrated the potential utility of this target in multiple disease areas, such as sepsis, fibrosis, chronic obstructive pulmonary disease, cardiac hypertrophy, and male contraception; it accelerated proprietary programmes in industry, and it enabled the establishment of many new biotechs [19]. In the five years since the release of the first BRD4 chemical probe, multiple companies have developed proprietary molecules against the target and are testing them in more than 15 clinical studies [22, 23]. This example provides a remarkable demonstration for how a freely available, high-quality, novel probe can catalyse science, drug discovery, and enterprise. Other BET family members have also been implicated in cancer. For example, the components of the chromatin remodelling switch/sucrose non-fermentable (SWI/SNF) complex are recurrently mutated in tumours, thereby suggesting that altering the activity of the complex plays a role in oncogenesis [24]. A team from industry and academia has now designed an in vivo active inhibitor of the BET family member BRD9, a component of the SWI/SNF complex BI-9564 [25]. The selective small molecule inhibitor is well tolerated and has appropriate pharmacokinetic properties for it to be used to explore the function of BRD9 in disease models [26]. The SGC has now made this chemical probe freely available at http://www.thesgc.org/chemical-probes to the scientific community to explore BRD9 biology.

Because open initiatives can reinvigorate the drug discovery process, it is important to consider what we can learn from examples like the ones above and how we can apply those lessons to improve target discovery and selection pipelines. First, we need to create the right sort of precompetitive PPP—one that has the appropriate resources, scientific expertise, and oversight to prioritise suitable genes and targets, elucidate their protein structures, and generate robust, cell-permeable chemical tools to enable the interrogation of biological and disease networks [27]. The pooling of resources in such a PPP can catalyse the exploration of new biology and chemistry, as well as efforts in areas currently deemed to be intractable, difficult, or high risk. Selecting a drug target that has genetic data supporting its role in a relevant disease can double the success rate in clinical development; thus, it is important to use genetic information to prioritise suitable genes and target candidates [28]. In addition, the prioritisation of new targets for lead optimisation or drug discovery efforts also relies on translational assays that are predictive of clinical outcomes. These assays depend on access to patient-derived materials [29], which are more readily accessed in academic or clinical environments, where the patients are located. Assays comparing cells or tissues from healthy humans and patients with a disease will provide insights into patient heterogeneity, help to identify the best subsets of patients for initial clinical proof-of-concept studies, and, most importantly, identify the best targets for early drug discovery. Another ingredient critical to building an impactful, precompetitive PPP is open access (i.e., data and reagents are made available to the world and not just to the consortium of funders) to unencumbered outputs (i.e., no restriction on their use). Rapid public access to the data and knowledge will also reduce unnecessary duplication and wastage of efforts. Many PPPs, however, struggle with the concept of sustainability. How can a consortium ensure that the results and tools generated during the funding period remain available to the scientific community after the funding has expired? One way is to make these reagents available through secondary distributers, such as vendors. Another is to ensure that the results are published not only in scientific journals, but also in independent, community-driven resources, such as The Chemical Probes Portal [30]. By combining commercial and academic incentives and resources in PPPs, we can improve the quality and reproducibility of the science pursued [27]. Overall, an impactful PPP is one that helps to convert creative ideas into practice by providing a reproducible and reliable framework in the form of tools, assays, and protocols for exchange and hypothesis testing. Such an approach allows the scientific community to work on a wide breadth of targets but, ultimately, lets the pharmaceutical industry choose the ones with which to make the huge investments in the discovery and development processes necessary to translate any given target into an approved therapy. This handoff from a precompetitive to a competitive model ensures the necessary return on investment required by industry to pursue regulatory approval. Although we do not anticipate major short-term cost benefits from crowdsourcing, we should see an overall increase in productivity because there should be fewer failures due to an improved understanding of disease biology and better project prioritisation.

## Harnessing the scientific crowd requires a reliable framework

**Fig 2:**
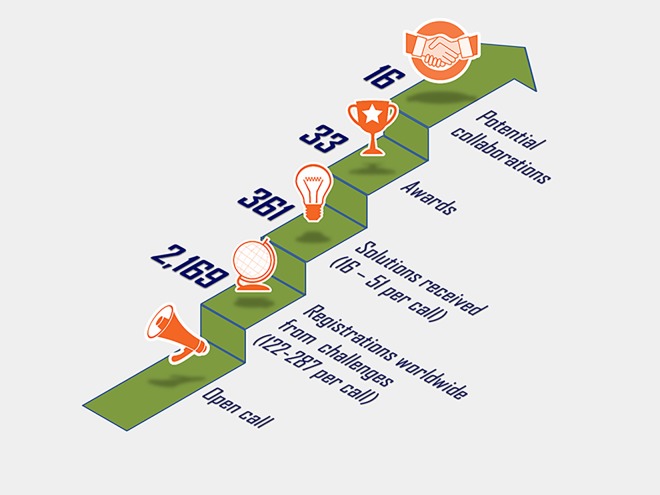
Attrition numbers for InnoCentive challenges from solver registration to potential collaboration. Boehringer Ingelheim has instigated 12 open crowdsourcing calls with InnoCentive that cover a wide range of topics, ranging from studying new translational models of psychiatric disease, to new approaches for the in vivo modulation of gene expression in lymphocytes, and to mimicking smooth muscle remodelling in severe asthma. A total of 2,169 solvers have registered themselves at the InnoCentive website in response to these calls and have provided more than 361 solutions, of which 33 have won awards, and 16 potential collaborations have been identified (status as of 12 December 2016).

Even if a precompetitive PPP can achieve all of the goals outlined above, this discovery system will remain less than optimal. An essential ingredient is still missing, namely, the scientific crowd. “Crowdsourcing” was first coined by Jeff Howe [31]; he defined the term as, “the act of taking a job traditionally performed by a designated agent and outsourcing it to an undefined, generally large group of people in the form of an open call,” or, more simply, “the application of open source principles to fields outside of software.” Again, we see a parallel to selective revealing in the software industry. Today, we recognise that crowdsourcing is a form of sourcing in which individuals or organisations solicit contributions from Internet users to obtain desired services or ideas. Many pharmaceutical companies have become adept at defining problems or questions that need to be solved, and they often reach out to the wider scientific community for solutions. A pharmaceutical company typically either establishes its own Internet portal to solicit solutions from potential solvers, as is the case with GlaxoSmithKline (GSK) or Bayer, or works with specialist brokers who have platforms, such as InnoCentive, NineSigma, or Kaggle [32]. Indeed, the crowdsourcing platform, Grants4Targets, has triggered or generated relevant input into ten drug discovery projects at Bayer [33]. Boehringer Ingelheim has pursued several crowdsourcing projects with InnoCentive that cover a wide range of topics, ranging from studying new translational models of psychiatric disease, to new approaches for the in vivo modulation of gene expression in lymphocytes, and to mimicking smooth muscle remodelling in severe asthma. A total of 2,169 solvers have registered at InnoCentive in response to these calls and have provided more than 361 solutions, of which 33 have won awards, and 16 potential collaborations have been identified (Fig 2). However, these approaches suffer from one major drawback: innovation requires that a creative idea be reduced to practice in the form of an actionable plan. For example, in the case of InnoCentive-like calls, the challenge is to negotiate the individual bilateral agreements and assemble the teams required to implement the identified solutions. The process of converting an idea into action is by necessity an iterative process that needs to be collaborative and free from encumbrance (i.e., a team must be free to pursue solutions in a way that does not restrict its freedom to operate). To maximise the scientific crowd’s freedom to operate, the SGC has chosen to make its chemical probes freely available via its website and via commercial vendors. The scientific crowd will be able to more deeply and quickly explore and disseminate new biology (i.e., potential new insights about potential drug targets) when it has access to well-characterised chemical tools.

Bringing scientists together with ideas and skills at one site and providing them with access to an academic infrastructure and equipment are another way of helping to reduce creative ideas to practice. The BioMed X Innovation Center has taken this approach to match crowd-driven problem-solving with implementation. Here, the pharmaceutical industry works with BioMed X to define a medical challenge; they then reach out to early career scientists, inviting them to supply potential solutions and apply for a position in a group that will be formed to take on the challenge. The requests typically receive about 400–600 responses from around the world, of which BioMed X picks about 15 of the most promising concepts submitted and invites the candidates to Heidelberg for an intense five-day competition [7]. After this competitive selection process, the chosen team of scientists then works exclusively on the scientific challenge with supporting infrastructure and in close association with mentors from industry and academia in an iterative process. Funders have the option to acquire all intellectual property rights, and the academic scientists have the right to publish their results. Thus, this approach combines a crowd-outreach component and an action plan component that are linked by the funding of a dedicated team and infrastructure. One of the first teams to go through this four-year exercise created bioinformatics tools for designing highly selective inhibitors of kinases, and the pharmaceutical sponsor, Merck KGaA, has already acquired the intellectual property rights and licensed them back to the team to form a start-up [7]. BioMed X has also worked with Boehringer Ingelheim to successfully apply this approach to establish a team of young scientists in Heidelberg who are working to identify key epigenetic regulators of chronic obstructive pulmonary disease [34].

The question of the underlying business model for a crowdsourcing platform is an interesting one (Table 1). Several companies such as Kaggle, NineSigma, or InnoCentive have developed Internet platforms and sell a service as part of their business. In contrast, some pharmaceutical companies such as GSK or Bayer have developed their own platforms in order to access solutions. Finally, many nonprofit organisations have developed crowdsourcing approaches as part of PPPs. All of them provide solvers with a reward in some form or other, but, interestingly, it is not always monetary. Sponsors of crowdsourcing calls often work with service providers to reward solvers with financial prizes so that they, the sponsors, can use the submitted ideas internally. Alternatively, the pharmaceutical industry can fund a bilateral collaboration with the solver to progress the idea further or can provide the chance to work with a team of like-minded scientists supported by a scientific infrastructure, as established by BioMed X. These approaches all have one thing in common, namely, the need for dedicated resources and personnel to review the proposed solutions and reduce the creative idea to practice. The reward for precompetitive PPPs such as the SGC is access to a high-quality chemical probe that enables an impactful publication or new results that trigger a new drug discovery project. Finally, we should also not underestimate the power of peer recognition, which was one of the main driving forces behind the development of GNU/Linux.

**Table 1:** A comparison of the different biomedical crowdsourcing approaches.

Crowdsourcing approach	Examples	Potential reward
Precompetitive PPP	SGC, Single Nucleotide Polymorphism Consortium, International HapMap Project, Innovative Medicines Initiative	Sharing of results, scientific recognition, early access to tools and biomarkers, shared methods and standards
Commercial platforms	Kaggle, NineSigma, InnoCentive	Successful solvers receive monetary prizes, potential for bilateral collaborations
Innovation centre or incubator	BioMed X	Employment, laboratory, and supporting infrastructure in an innovation centre
Corporate pharmaceutical platforms	GSK (Discover Fast Track) Bayer (Grants4Targets)	Successful solvers receive monetary prizes, potential bilateral collaborations, and/or access to drug discovery resources at the pharmaceutical company

Biomedical research is evolving rapidly. Open innovation has become a fundamental part of the drug discovery process. Bilateral partnering agreements between companies and academic investigators will continue to provide important starting points for drug discovery projects. However, precompetitive PPPs are playing an ever-increasing role in the process because they bring together the best academic and industrial scientists in an open environment in which they can share solutions and tools that are unencumbered by operational restrictions. Crowdsourcing as a means of selectively revealing problems to encourage problem-solving is also contributing to drug discovery research. However, the real benefit of both of these open-innovation approaches can only be achieved when the power of enabling independent scientific crowds can be fully harnessed by converting creative ideas into practice with actionable plans and tools for the discovery of innovative medicines.

## Abbreviations

BET: bromodomain and extra-terminal
GNU: GNU's Not Unix
GSK: GlaxoSmithKline
OS: operating system
PPP: public–private partnership
R&D: research and development
SGC: Structural Genomics Consortium
SWI/SNF: switch/sucrose non-fermentable
